# 3D Morphometric Analysis of Human Primary Second Molar Crowns and Its Implications on Interceptive Orthodontics

**DOI:** 10.3390/ijerph18126201

**Published:** 2021-06-08

**Authors:** Alessandro Nota, Vincenzo Quinzi, Federico Floriani, Clizia Cappelli, Simona Tecco, Giuseppe Marzo

**Affiliations:** 1 Dental School, Vita-Salute San Raffaele University and IRCCS San Raffaele, 20132 Milan, Italy; nota.alessandro@hsr.it; 2 Department of Life, Health and Environmental Sciences, University of L’Aquila, 67100 L’Aquila, Italy; vincenzo.quinzi@univaq.it (V.Q.); giuseppe.marzo@cc.univaq.it (G.M.); 3 Private Practice, 20122 Milan, Italy; fede.flo@alice.it (F.F.); clizia.cappellic@gmail.com (C.C.)

**Keywords:** interceptive orthodontics, human teeth, orthodontic removable appliances, orthodontic appliances, orthodontic bands, digital orthodontics

## Abstract

The second primary molar represents an anchorage element in interceptive orthodontics. The present study aims to analyze the 3D morphology of primary second molars in order to provide reference data and implications about the development of orthodontic bands for second primary molars. Digital models of dental arches from 150 subjects in primary or mixed dentition were analyzed. Six dimensional variables were digitally measured for each second primary molar, and the mean and standard error of the mean (SEM) were calculated and compared applying Student *t*-test statistical analysis. The mean value results show statistically significant dimensional differences between the upper and lower teeth, (mostly *p* < 0.0001), except for the variable h1, while only the variable h1 showed significant differences between the antimetric teeth (left and right). The dimensional variations between the right and left molars were considerably minor compared to those found by comparing the upper and lower arches. A significantly higher dimension of the lower molars and a more rectangular shape were observed.

## 1. Introduction

Interceptive treatment is fundamental in modern orthodontics [1,2]. In fact, intercepting a dental or skeletal problem and solving it while the patient is still growing can allow for the avoidance of orthodontic extractions or, in severe cases, surgical orthognathic treatment or temporomandibular joint involvement [3,4,5]. In order to obtain a good orthopedic effect, it is necessary to make a correct diagnosis, choose the right device, and start the treatment at the right time, which can often coincide with the period of mixed dentition. One of the primary problems when orthodontic devices are placed in mixed dentition is related to their anchorage [6]. Consequently, it is important to use orthodontic devices that guarantee considerable reliability and precision to minimize dental compensations or undesired dental movements in order to reduce the problems of debonding of the devices, discomfort, and pain related to a non-perfect adhesion or ergonomics [7].

In addition, it is preferable to avoid dental extrusion with a consequent increase in the vertical dimension, root resorptions, tooth–alveolar compensation such as buccal tipping, or periodontal problems, as all of these aspects can determine loss of effectiveness and efficiency of orthodontic treatment [8].

On the other hand, the use of primary elements as anchors instead of the first permanent molars can protect the latter from the risk of demineralization and carious pathology. For example, it should be noted that the increasing prevalence of MIH (molar incisor hypomineralization) in the pediatric population can only sporadically affect the second primary molars, making the use of these teeth as anchorage rather than the first molars an interesting approach when interceptive treatment with fixed devices is necessary [9,10]. For all these reasons, with increasing frequency, orthodontic devices, such as the rapid palatal expander, the extraoral traction, the Delaire mask, the lip bumper, and space holders, are anchored by orthodontic bands to the second primary molars [4,11,12].

According to the above, it is clear that if the first permanent molar represents, in the development of human dentition, the key element of occlusion, the second primary molar could represent a key element in interceptive orthodontics [11].

Currently, the request for orthodontic bands for primary molars is not met by an adequate offer from manufacturers. In fact, there are no orthodontic bands specifically designed for these teeth on the market. For this reason, bands made for lower sized permanent teeth or preformed metal crowns are often used to weld the remaining equipment. Although this technical stratagem allows one to face most cases without great difficulty in daily practice, it is true that the morphology of the primary molars, in particular that of the second primary molars, may not be ideal, often leading to a series of disadvantages, such as the deception of orthodontic devices or the need for adaptation of the bands by the dental laboratory in order to ensure good stability and adhesion of the teeth [9].

In some cases, instead of adapting the bands to the primary molars, the reverse procedure could be used in which they are adapted to the bands by using additive odontoplasty with composite materials or reduced by appropriately milling them with diamond cutters.

It could be beneficial to have orthodontic bands for second primary molars designed according to their size but also to their proper shape, which is not exactly similar to that of permanent molars, and this could be achieved with a specific band set or by using new 3D technologies for building CAD/CAM-customized primary molar bands. The present study aims to digitally analyze the 3D morphology of primary second molars in order to provide reference data and implications about the development of orthodontic bands for second primary molars.

## 2. Materials and Methods

For the present study, digital models of dental arches from 150 Italian subjects in primary or mixed dentition (75 males, 75 females; age range 6–11 years), all obtained using an intra-oral Scanner 3Shape D800 (3Shape A/S, Copenhagen, Denmark), were used. All of the impressions were performed by the same operator.

Inclusion criteria for the initial sample were: Caucasian race of the subject, completely erupted teeth, digital models obtained from good quality optical impressions, presence of the four second primary molars.

**Figure 1:**
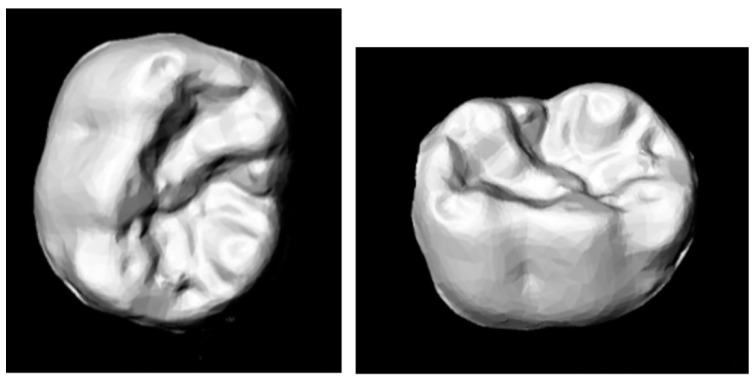
Digital reconstruction of the primary second lower molar.

**Figure 2:**
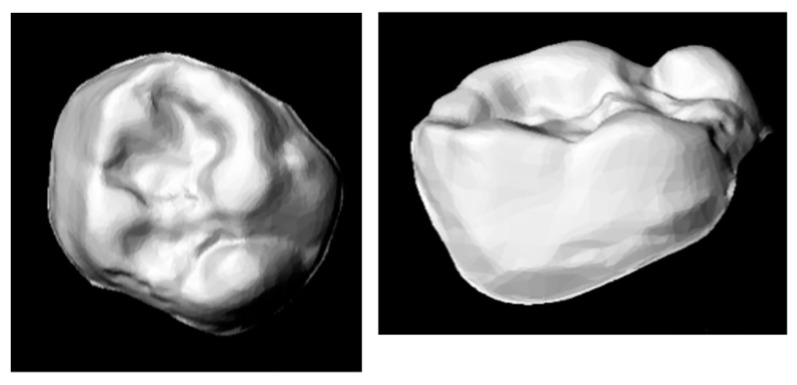
Digital reconstruction of the primary second upper molar.

Then, from a total of 600 teeth, the following exclusion criteria were applied: interproximal caries, dental wear, coronal fractures, erosion or cervical caries, ongoing orthodontic treatment, undererupted teeth. After applying the exclusion criteria, a total of 286 second primary molars were finally included in the sample: 70 upper right primary molars, 69 upper left, 73 lower right, and 74 lower left (Figure 1 and Figure 2).

All of the teeth were digitally measured by the same operator expert using Orthoanalyzer™ software (3Shape Orthosystem packet, 3Shape A/S, Copenaghen, Denmark) after a calibration period where she conducted several measurement tests for the study variables. To evaluate the intra-operator method error, the measurements of 20 dental casts were performed for a second time by the same operator after two weeks, and the intraclass correlation coefficient (ICC) was calculated showing values higher than 99.7.

**Figure 3:**
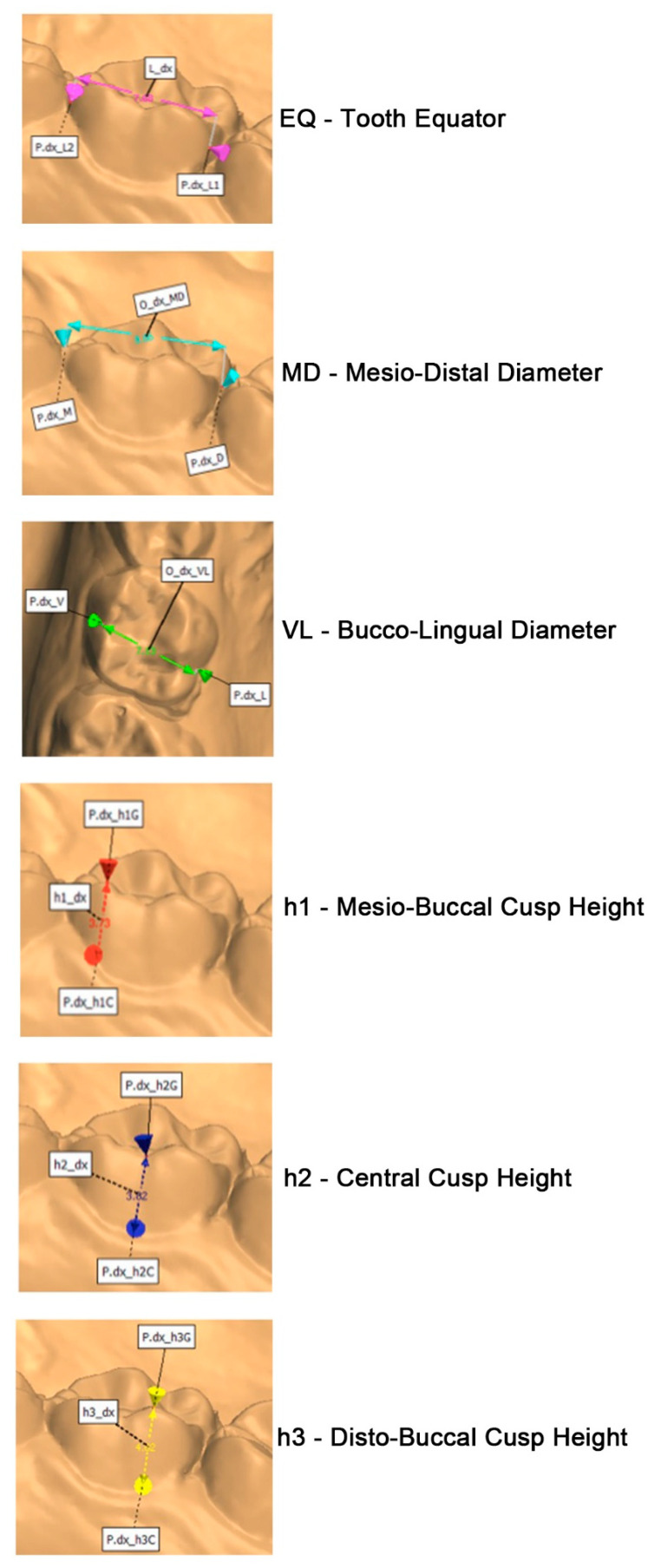
Digital measurements performed.

The following measurements shown in Figure 3 were needed for a complete morphologic analysis and as reference for orthodontic bands development, and they were calculated in the upper and lower arches. 

Tooth equator (EQ): This is considered as the maximum circumference taken on the buccal face of the tooth [13].

Mesiodistal diameter (MD): For a long time, the authors measured the distance between the marginal ridges because the contact points were not reachable. From an anthropometric point of view, the correct parameter to be measured, which was used in this study, is the distance between the points of contact with neighboring teeth measured parallel to the occlusal plane [8].

Buccolingual diameter (VL): This represents the maximum diameter of the crown from the beginning of the intercuspidal sulcus of the buccal surface to the beginning of the intercuspidal sulcus of the lingual/palatal surface [14].

Mesiobuccal cusp height (h1): The distance between the cervical line and the MD cusp is considered [15].

Central cusp height (lower molars)/central fossa height (upper molars) (h2): This is the measurement between the cervical tooth line and the central cusp for the lower molar and the deepest point of the sulcus between the tooth cusps for the upper molar [15].

Distobuccal cusp height (h3): The distance between the cervical line and the distobuccal cusp is considered [15].

The reference plane used in this study is identified through three points taken on a single tooth: the first on the distobuccal cusp, the second on the mesiobuccal cusp, and the third on the mesiolingual cusp.

### Statistical Analysis

In order to quantify the differences of the measurements between the upper and lower arches, for each variable (equator, mesiodistal diameter, buccolingual diameter, heights) the mean, standard deviation (SD), and standard error of the mean (SEM) of the values of the upper and lower primary molars (right and left) were calculated and compared. Considering that a Kolmogorov–Smirnov test confirmed the normality of the distribution, a Student *t*-test for independent samples was applied for all of the comparisons.

The tests were performed with StatPlus software (AnalystSoft Inc., Walnut, CA, USA), and the significance threshold was set at 0.05.

## 3. Results

Table 1 and Table 2 summarize the mean values and SD results showing statistically significant dimensional differences between the upper and lower teeth, (mostly *p* < 0.0001), except for the variable h1, while only the variable h1 showed significant differences between the antimetric teeth (left and right).

Overall, the dimensional variations between the right and left molars were considerably minor compared to those found by comparing the upper and lower arches.

## 4. Discussion

In the present study, a morphometric analysis of primary second molars was performed. The results showed that there are significant differences between the upper and lower primary second molars, and, in particular, the greatest dimensional differences were found mainly at the tooth equator and at the mesiodistal diameter. Notably, the equator of the lower arch is approximately 1.1 mm longer, while the mesiodistal diameter is about 0.8 mm longer. The minor discrepancy was recorded at the buccolingual diameter of about 0.3 mm.

In the present study, the mesiodistal diameter resulted the longest parameter, despite the fact that in some studies, the buccolingual diameter has been observed to be longer than the mesiodistal diameter, especially in Caucasian ethnic groups. This is reported to be due to the presence of the Carabelli cusp, which is often particularly developed in the primary teeth [16,17].

In general, all measurements are higher in the lower molars than the upper ones, except for the distobuccal cusp height (h3); thus, it can be concluded that the lower primary second molars are larger than the upper primary molars, as already demonstrated in the literature [18,19,20].

In the cross-section, the second upper primary molar has a more squared shape, as the diameter of the mesiodistal tooth differs slightly from the buccolingual diameter, while the second lower molar shows a dimensional discrepancy of about 2 mm between these two parameters; thus, the lower primary molars have a more rectangular shape.

While the mesiodistal diameter of the tooth is related to the height of the sagittal section, the lower molar assumes a trapezoidal shape with a decreasing trend towards the buccodistal cusp. The h3 parameter shows the lowest average value. Differently, the upper primary molar, on a sagittal plane, assumes a more rectangular shape, as the discrepancy among the heights of its three cusps is minimal.

In the literature [18,19,20], there are some previous studies that analyzed the shape and dimensions of the primary teeth in other populations, also comparing them with those of the first permanent molars and considering almost the same measurements of the present study, except for the tooth equator, which was analyzed in the present study for the first time. Furthermore, no previous studies were performed on the Italian population.

To the best of the authors’ knowledge, this is also the first study in which calculations of the measurements were performed with a digital workflow on 3D files obtained by intraoral optical scans. Previous studies showed the accuracy of digital technologies for dental measurements [21,22].

Reviewing the anthropological literature on odontometry, there are several methods for recording the main dental dimensions; there are many detractors of the impossibility to carry out comparative studies among human populations on the basis of dental data, as in tooth-to-tooth comparison, it is not possible to transcend intrapopulation variability.

Bailey, in 2004, identified limitations in the methodological approach previously used for morphometric surveys [23]. The teeth, in fact, even more than other skeletal districts, have a complex and irregular shape, on which it is difficult to standardize reference points to proceed with measurements, as claimed by Hillson [15], and for which it is even more evident that the limited measurements that can be carried out cannot account for the total complexity of the tooth itself.

In addition, even in the encoded measurements, there are anatomical points of reference to perform the measurements, and the given different interpretations only increase the possibility of error between different operators. In addition, the references used for the correct measurement are often subject to the influence of tooth orientation in space and, at the same time, to the impossibility of verifying the relative orthogonality between diameters. Therefore, in this scenario, the use of digital scans—adopted in the present investigation—could help to perform the measurements more accurately.

Finally, but of fundamental methodological importance, is the presence of wear. Dental wear can occur, depending on the type of tooth in question, in different areas and with different intensities, thus affecting correct measurement of the tooth. the dental region most affected by wear is usually the occlusal surface, rendering impractical the relief of crown height. Moreover, the wear of the occlusal surface, despite rarer, may even compromise the actual size of the buccolingual diameter. As for the mesiodistal diameter, it is in most cases altered by the presence of interproximal facets of wear. For these reasons, in the present study, the presence of dental wear on the second primary molars was considered as exclusion criterion.

A problem highlighted in the literature is the lack of specific homogeneity in the nomenclature attributed to dental measurements. This deficiency was in part filled by the literature contribution that limits the attribution of the qualification of diameters to two measures, avoiding the misunderstanding that other terms would involve as well as the inclusion of the appraisals of other skeletal districts. Despite several attempts, the management of the nomenclature remains unclear today [15].

Given the complexity of dental forms, there are different ways of recording these measurements in the literature. In this study, six dental measurements were carried out on each primary molar. It can therefore be concluded that there are several factors that can lead to measurement errors. In this study, we tried to limit these errors as much as possible by using digital software for dental measurement and with strict quality selection of the models included in the analysis.

The reliability and precision of 3D technology has been progressively demonstrated by several studies regarding its dental applications and in all fields of detection and reproduction of anatomical structures for diagnostic, therapeutic, and prosthetic rehabilitation.

The main advantage of using this method is that it is capable of making very precise measurements due to the ability to magnify the virtual model and to identify points for measurements, thus reducing the margin of error. Moreover, as there is no contact with the plaster model, there is no risk of damaging the model with a mechanical caliber and, therefore, of altering it.

Among the limitations, the identification of the anatomical points on the PC screen requires a training process, and the accuracy in the scanning of areas such as sub-frames and curved surfaces could influence the results.

Comparing the primary molars and the permanent ones both from a dimensional and morphological point of view, it is obvious that the second primary molars have smaller dimensions than the permanent ones, but it is not so easy to exactly identify the 3D morphological differences between the two sets of teeth without appropriate morphological analysis. In fact, as previously demonstrated, the upper primary molars have a more squared and squat shape in occlusal vision; the upper permanent molars in the cross-section appear to have a more rectangular shape with longer sides in the buccolingual sense.

As for the lower arch, always considering the cross-section, the permanent molar has a more squared shape as opposed to the primary one, which instead has a rectangular shape with long sides in the direction of the mesiodistal diameter.

On the basis of these considerations, it could be observed that the orthodontic bands built for the first permanent molars do not guarantee optimal precision for the fitting of the primary molars. Reporting on the dimensional and morphological differences found as well as the design of orthodontic bands specifically for primary molars, we discuss below the rendering of bands for primary and permanent molars. However, to delineate the precise shape of the crown of the primary second molars, further measurements, such as analysis of the average inclination of all four tooth faces, are needed. This could represent a limitation of the data of the present study for the purpose of primary molar bands production. Another limitation is also that primary molar measurements were compared with those of permanent molars with reference to the previous literature and without directly performing measurements on this set of teeth.

In the final analysis, to create an ideal band set that fits perfectly to the primary molars in shape and size, for the six measurement parameters of each tooth, the maximum, minimum, and medium values should be considered in order to outline a type of shape and a range of measures that can then inspire the production of a set of ideal bands. The development of digital technologies and intraoral scanners could help in overcoming the individual variability between primary molars by producing customized bands and orthodontic devices that can provide more precise orthodontic outcomes via ideal fitting with primary molar anatomy. From this point of view, this could represent a solution to the creation of another set of molar bands, though standard band sets are usually cheaper. Nevertheless, this requires 3D technology that is still not available in each dental office and laboratory.

In order to design a set of bands for second primary molars, the equator measurement was also added to the other measurements in the present study. This has previously been applied studies in order to allow for a proper band shape and retention. Furthermore, the difference observed between the different second primary molars (upper/lower, right/left) confirms the fact that different bands are needed for the upper and lower arches.

In order to obtain bands with perfect adaptation to the exact polyhedral shape of the upper and lower primary molars, or in the case of particular morphology or considerable differences between antimetric teeth (also for the variable h1), currently, the only solution is the use of recent digital technologies that allow for the designing and melting of customized bands for each patient, which could result in achieving ideal fitting and retention for interceptive orthodontic appliances with anchorage on the primary dentition, such as the rapid palatal expander, Delaire mask, lip bumper, and space maintainers.

**Figure 4:**
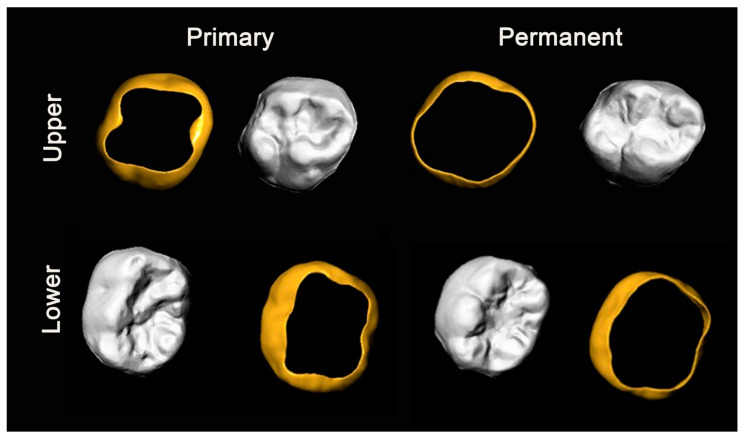
Morphological comparison between primary and permanent molars and the design of the respective orthodontic bands.

**Figure 5:**
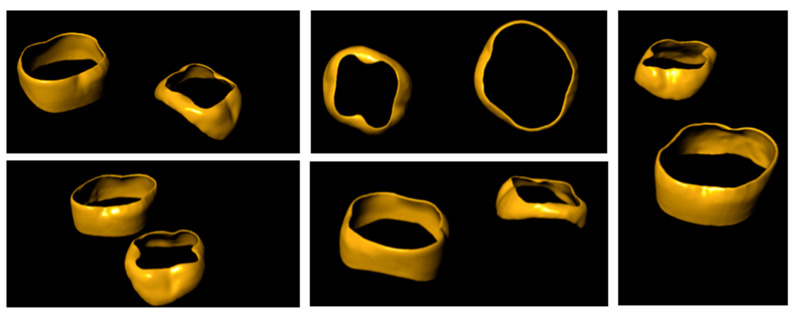
Multiperspective comparison between the proposed design of orthodontic bands for (upper and lower) primary and permanent molars.

Figure 4 and Figure 5 highlight the differences between the two sets of teeth that are essential for optimal adaptation of the bands and a solid anchorage for orthodontic devices.

## 5. Conclusions

The morphometric analysis of primary second molars in the Italian population showed a significantly higher dimension of the lower ones, characterized by a more rectangular shape, and dimensional differences were found mainly at the tooth equator and at the mesiodistal diameter. In the present study, the mesiodistal diameter had the longest parameter, a result different from those of previous studies that found the buccolingual diameter to be longer than mesiodistal, especially in Caucasian ethnic groups.

The data of the present study are principally generalizable to the Italian population, but previous studies in other populations apparently provided similar results.

## Figures and Tables

**Table 1:** Descriptive analysis of data.

ELEMENT Nr. (FDI Notation)	Measure (mm)	Variables					
		EQ	MD	VL	h1	h2	h3
55	Mean	7.55	8.58	7.53	4.04	4.20	4.13
	SD	0.68	0.64	0.70	0.60	0.54	0.58
	SEM	0.08	0.08	0.08	0.07	0.06	0.07
65	Mean	7.52	8.50	7.54	3.98	4.12	4.13
	SD	0.62	0.67	0.71	0.63	0.56	0.70
	SEM	0.08	0.08	0.09	0.08	0.07	0.08
75	Mean	8.75	9.32	7.25	4.22	4.64	3.04
	SD	0.63	0.57	0.61	0.70	0.55	0.58
	SEM	0.07	0.07	0.07	0.08	0.06	0.07
85	Mean	8.68	9.35	7.22	3.78	4.71	3.37
	SD	0.62	0.63	0.66	0.76	0.54	0.80
	SEM	0.07	0.07	0.08	0.09	0.06	0.09

SD: Standard Deviation, SEM: Standard Error of the Mean.

**Table 2:** Student *t*-test statistical comparisons.

VARIABLE	EQ	MD	VL	h1	h2	h3
Upper/Lower	<0.0001	<0.0001	0.0002	n.s.	<0.0001	<0.0001
Right/Left	n.s.	n.s.	n.s.	0.01	n.s.	n.s.

## Data Availability

The data that support the findings of this study are available from the University of L’Aquila, but restrictions apply to the availability of these data, which were used under license for the current study and so are not publicly available. Data are, however, available from the authors upon reasonable request and with permission of the University of L’Aquila partner.
